# Educational Impact of an Instagram Account on Histology Teaching: Mixed Methods Approach in Medical Education

**DOI:** 10.2196/89044

**Published:** 2026-10-16

**Authors:** Fatéméh Dubois, Damien Ambrosetti, Lucie Tosca

**Affiliations:** 1 Department of Pathology, Caen-Normandie University Hospital, Caen-Normandie University Medical School, Caen, France; 2 Department of Pathology, Nice University Hospital, Côte d'Azur University Medical School, Nice, France; 3 Department of Histology Embryology and Cytogenetics - Genomic Medicine and Pharmacogenomics, Paris-Saclay University Medical School, APHP.Hôpitaux Universitaires Paris Saclay, 78, rue du Général Leclerc, Le Kremlin Bicêtre, 94270, France

**Keywords:** medical education, social-media learning-based, Instagram, histology, embryology

## Abstract

**Background:**

Morphology-based disciplines such as histology, embryology, and cytogenetics require strong visual learning and rely heavily on visual pattern recognition, with more complex integrative processes, that is, higher-order cognitive skills that connect, interpret, and apply knowledge across contexts. These skills may be supported by social media formats. In parallel, medical students increasingly engage with digital and social media platforms, prompting interest in their potential educational value. Instagram (Meta Platforms, Inc), as an image-centered and interactive medium, has emerged as a potential pedagogical tool. The Histo.Embryo Instagram account was launched in 2023 under the auspices of the French National College of Histologists, Embryologists, and Cytogeneticists (CHEC) to complement traditional teaching with bilingual, interactive, image-based posts.

**Objective:**

This study aimed to evaluate the pedagogical contribution of the Histo.Embryo Instagram account with a focus on students’ engagement, usage patterns, perceived learning outcomes, and qualitative user experience. This learning platform was developed as a visual and interactive tool to complement traditional teaching in histology, embryology, and cytogenetics.

**Methods:**

A mixed methods design combined a structured online survey (N>100), Instagram analytics (90-day period), and qualitative feedback. Quantitative data assessed usage frequency, preferred content formats, perceived utility, and self-reported learning outcomes. Qualitative data were analyzed thematically to explore how students interacted with and integrated the content into their learning practices.

**Results:**

Instagram was the dominant platform among students, with 81% (101/125) reporting daily use. The account reached 2582 unique users and 168,778 views. Static quiz-based posts generated 96% (203/207) of engagement, highlighting a strong preference for active recall formats. Survey data showed that 98% (122/125) perceived improved understanding, 64% (80/125) enhanced visual recognition, and over two-thirds reused content in academic settings. Qualitative findings provided additional insight into these patterns. Students described the content as accessible, visually clear, and aligned with exam expectations, emphasizing its role in simplifying complex concepts and supporting memorization. Many reported using posts for rapid revision, self-assessment, and consolidation of prior knowledge, suggesting integration into self-regulated learning strategies. The interactive quiz format was perceived as motivating and cognitively engaging.

**Conclusions:**

The Histo.Embryo Instagram account suggests that Instagram, when intentionally structured, may complement traditional teaching by supporting visual learning, active recall, and flexible study practices. Nevertheless, these findings should be interpreted cautiously due to potential self-selection bias, reliance on subjective measures, and the absence of controlled comparisons. Further research is needed to evaluate its impact on objective learning outcomes and long-term knowledge retention.

## Introduction

Teaching in morphology-based disciplines such as histology, embryology, and cytogenetics has traditionally relied on visual resources (such as histological slides, diagrams, and illustrative materials) and is rooted in a pedagogy that emphasizes observation, repetition, and memorization [1,2]. These 3 disciplines are part of the core subjects taught during the preclinical years of medical studies. Histology focuses on the study of the structure of cells, tissues, and organs in order to establish structure-function relationships. Embryology is devoted to the study of embryonic development, that is, the period of life between fertilization and birth. Cytogenetics, for its part, is the discipline concerned with the study of chromosomes and related pathologies. In recent years, the digital habits of health students have evolved considerably, prompting educators to rethink their teaching approaches [3,4]. Against this backdrop, social media has emerged as a potential tool for medical education.

Instagram (Meta Platforms, Inc) is increasingly recognized and used as a tool to support learning in medical and dental education, particularly among students who are already frequent users of the platform [5-7]. Recent studies also suggest a shift in students’ preference from older platforms such as Facebook (Meta Platforms, Inc) and Twitter (subsequently renamed X; X Corp) toward more visual and mobile-first platforms like Instagram [6,7]. Notably, Instagram’s core user base, individuals aged 18 to 34 years, corresponds closely with the age demographic of most medical students [8]. Instagram’s strength lies in its visual nature (photos, videos, stories, and reels), making it well-suited for teaching visually dominant subjects commonly found in medical and dental curricula such as anatomy, histology, and pathology [1,2,6,9-12]. The platform enables the sharing of a variety of educational materials, including annotated clinical or histological images, short videos, interactive quizzes, and links to additional resources [1,2,6,13].

Due to its mobile-first design, Instagram integrates seamlessly into students’ daily lives, even during informal moments such as commuting or breaks, thus supporting more continuous and contextualized learning [8,10,14]. It also encourages conversational interaction and peer-to-peer exchange through likes, comments, and shares [5,8,11], fostering a sense of community among learners. Students can engage with content actively, ask questions, and share insights, which strengthens their connection to both peers and educators [5,11]. The platform’s ability to bridge formal and informal learning contexts has also been highlighted, helping to reduce the perceived distance between teachers and students [7,15]. Some studies have emphasized the educational benefits of Instagram, including increased accessibility, support for microlearning and spaced repetition, and enhanced engagement among visual learners [2,10,12]. Indeed, one study examined the use of social media platforms to support learning in medical education and reported increased student engagement, particularly when content was structured in short, visually oriented formats aligned with curricular objectives [10]. Other authors highlighted how social media can facilitate knowledge reinforcement and promote active learning behaviors, especially when learners interact with content asynchronously [12]. Similarly, Escamilla-Sanchez et al [2] emphasized the role of digital platforms in fostering learner engagement and supporting informal learning alongside formal teaching, while also noting variability in active participation.

Reported outcomes include improved confidence, reduced stress, better understanding of visual concepts, and in some cases, higher academic performance [1,16]. Furthermore, Instagram appears to contribute to the formation of professional identity by enabling students to engage with the norms, practices, and values of their future professional communities [9,11]. However, these benefits depend on intentional pedagogical design. In the absence of a structured framework, Instagram may foster passive content consumption [6], fragmented attention [17,18], or exposure to unverified information [7,19]. The blurring of academic and personal spheres, issues around privacy and professionalism, lack of institutional guidelines, and the demands of content curation all represent challenges to its effective integration into coherent educational strategies [5,19].

It is within this context that the Histo.Embryo Instagram account was launched in 2023 under the auspices of the French National College of Histologists, Embryologists, and Cytogeneticists (CHEC). This collaborative project brings together around twenty educators from several French medical faculties, with a clear objective: to make histology, embryology, and cytogenetics more accessible, interactive, and aligned with the digital habits of health students. Weekly bilingual (French and English) posts feature annotated histological images, course summaries, clinical questions, and interactive quizzes. The initiative is unique in France and is designed to enrich traditional teaching by promoting the cocreation of concise, engaging, and visual learning materials across institutions.

This study aimed to evaluate the student engagement with educational content of the Histo.Embryo Instagram account, developed as a visual and interactive tool to complement traditional teaching in histology, embryology, and cytogenetics. The research followed a mixed methods approach, relying primarily on a structured survey composed of both closed and open-ended questions, on platform analytics drawn from Instagram account statistics, and on informal qualitative feedback gathered during histology laboratory sessions. From a quantitative perspective, the study sought to describe student demographics and digital habits, analyze usage patterns of the Instagram account, and assess students’ satisfaction and preferences regarding the different content formats. From a qualitative perspective, the study aimed to better understand students’ lived experiences and perceptions of learning with the Histo.Embryo Instagram account. Through this integrated approach, the study contributes to a more precise characterization of student engagement patterns and perceived usefulness of an Instagram-based educational initiative in morphology-based education, in alignment with the digital practices and expectations of today’s health students.

## Methods

### The Histo.Embryo Instagram Account

In the French medical curriculum, histology is taught as a fundamental basic science during the first 3 years of medical studies. It is integrated progressively from the first year through the second and third years of the preclinical cycle, often in conjunction with embryology and cytogenetics. Teaching formats vary by faculty but typically combine lectures, practical laboratory sessions with microscope slide analysis, and digital resources. Histology sessions are generally scheduled early in the semester to establish foundational knowledge, while more clinically oriented disciplines such as pathology or physiology are introduced later.

Recognizing the need to complement traditional teaching methods and to enhance student engagement in these core subjects, the Histo.Embryo Instagram account was created. This innovative program on Instagram was initiated by Caen University. The first educational post appeared in October 2023. The account was established as a public professional profile on Instagram, allowing unrestricted access to users across institutions and enabling the use of built-in platform analytics (Insights). It was regularly promoted during histology lectures in participating medical schools to encourage student engagement. To date, the account features 207 posts and nearly 2582 followers. This study was conducted subsequently, with data collection (survey and platform analytics) performed in 2025 over a defined observation period. The distribution of posts across histology, embryology, and cytogenetics was 186, 14, and 7, respectively.

The design of the account followed a collaborative and coordinated editorial strategy. A team of approximately 10 faculty members from different French universities contributed to content development. Posts were created in pairs (a primary author and a peer reviewer), ensuring both scientific rigor and pedagogical clarity. A core coordination team oversaw content validation, thematic diversity, visual formatting, scheduling, and publication.

Posts were typically published 2 to 3 times per week. Content was designed using standardized Microsoft PowerPoint templates and intentionally included a variety of educational formats, such as multiple-choice quizzes (MCQs), “zoom in and zoom out” image analysis, and caption-based questions, in order to address different learning strategies and maintain student engagement. However, content creation was collaborative and not constrained by predefined quotas or a fixed distribution across categories. As a result, the number of posts per category was not formally quantified, and variations in format distribution reflect the pedagogical choices and creative input of individual contributors. Visual material was sourced either from the CHEC image atlas or generated directly by the contributing educators. To ensure visual clarity and consistency, all images and explanatory text boxes were formatted in square layouts suitable for Instagram display. Posts were optimized for mobile viewing using standardized dimensions and fonts. Based on user feedback, posts were spaced out over the week to avoid overwhelming users and were often scheduled during high engagement times, based on platform analytics.

Each Instagram post was structured as a multislide bilingual publication, using Instagram’s carousel feature to present content in a clear, stepwise progression that guides students through the question, answer, and explanation phases. The first slide typically featured a histological image accompanied by an open-ended question in both French and English. In most cases, this was followed by one or two annotated slides providing the answer, with or without explanatory labeling. The subsequent slides delivered more detailed explanations, first in French, then in English, reinforcing key concepts. A final slide listed the names and institutional affiliations of the authors and coordinators, as well as the image source. The CHEC logo appeared on each slide to signal institutional endorsement and ensure visual continuity. Each post was also complemented by a caption containing thematic hashtags (eg, #histology, #histologielab, and #science) and tagged accounts of local student tutor groups across France (eg, @tutoratsantedijon and @tutsantecaen), thereby enhancing the visibility and dissemination of the educational content (see Figure 1).

**Figure 1.**
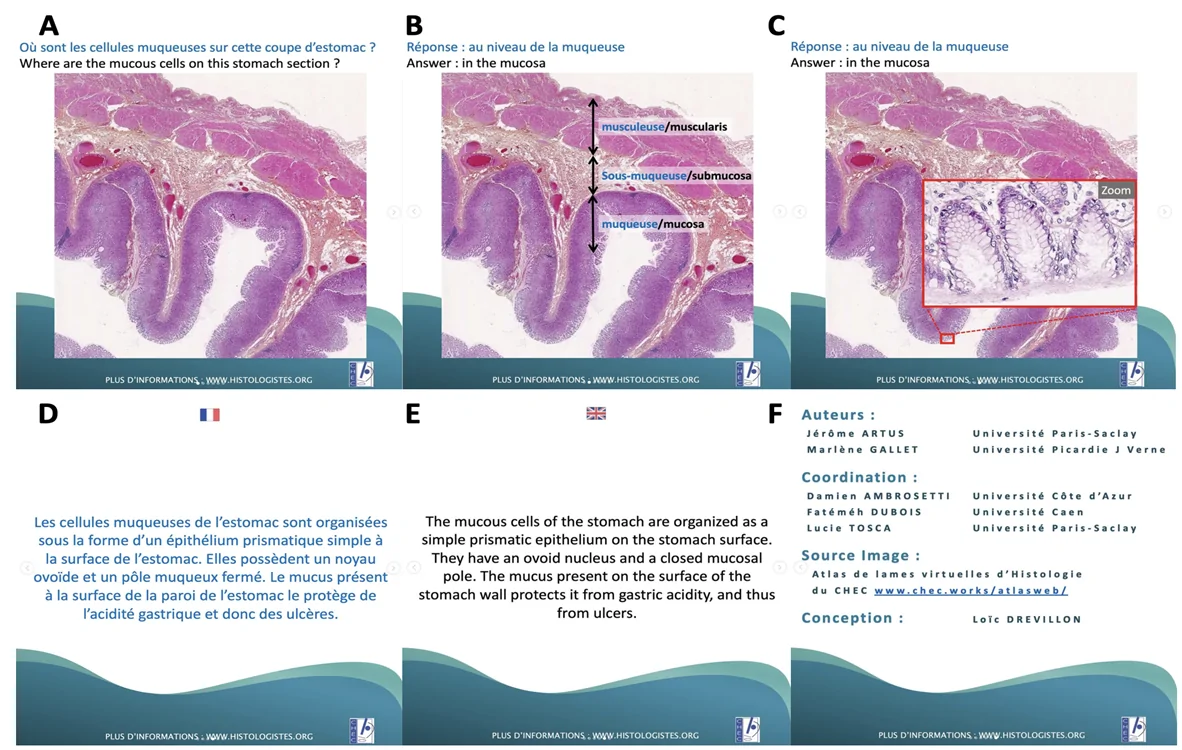
Example of a bilingual educational post published on the Histo.Embryo Instagram account. This multislide carousel illustrates the typical format used on the platform. (A) initial histological image with a guiding question presented in both French and English. (B and C) Labeled slides revealing the answer and highlighting key anatomical structures. (D and E) Explanatory text in French and English. (F) Final slide presenting the names and affiliations of authors, coordinators, and the image source. The College of Histologists, Embryologists, and Cytogeneticists (CHEC) logo appears on every slide to ensure visual consistency and institutional visibility.

Posts were deliberately aligned with the timeline of histology and embryology lectures and practical sessions. In some cases, additional posts extended beyond the curriculum to offer supplementary content for more advanced or curious students. Students could access the content asynchronously through their Instagram feed or by browsing the account’s full post library.

### Study Design and Participants

This study used a mixed methods design, combining quantitative and qualitative analyses to explore students’ engagement with and perceived educational value of the Histo.Embryo Instagram account. Quantitative and qualitative data were analyzed separately, and methodological triangulation was achieved during the interpretation phase to provide a broader understanding of usage patterns and students’ perceptions.

Data were collected from three complementary sources:

1. A structured online survey distributed through a publicly accessible link shared via Instagram posts (including closed and open-ended questions; 125 respondents) over a defined period (April to November 2025): Participation was voluntary and anonymous. Although respondents’ enrollment status was not formally verified, the survey was primarily directed at medical students attending histology courses
2. Informal in-person interactions with 30 students during histology laboratory sessions, consisting of spontaneous discussions rather than structured interviews
3. Aggregated analytics extracted from the Instagram platform (reflecting activity from 2582 accounts): Survey respondents and students participating in the informal discussions were recruited through convenience sampling, primarily among individuals who followed or interacted with the Histo.Embryo Instagram account.

### Quantitative Analysis

From a quantitative perspective, the study sought to describe student demographics and digital habits, analyze usage patterns of the Instagram account, and assess students’ satisfaction and preferences regarding the different content formats. It also aimed to evaluate students’ perceptions of the account’s usefulness in supporting their understanding, memorization, and ability to recognize histological structures, and to explore whether they had reused Instagram content in academic settings such as lectures, tutorials, or exams. In addition, quantitative platform data, including views, reach, content-type interactions, and engagement metrics, were analyzed to complement the survey results and provide an additional measure of account activity.

The quantitative component of the study was based on 2 complementary sources: structured online questionnaire and Instagram analytics. The questionnaire included closed-ended questions assessing usage frequency, preferred content formats, and students’ perceptions of the platform’s educational value, including its impact on understanding, memorization, and knowledge reuse in academic settings. Instagram analytics were extracted using the platform’s built-in Insights tool over a defined 90-day period (August 27 to November 27, 2025). Metrics included total views (impressions), reach (unique users), interactions (likes, comments, saves, and shares), and the number of engaging accounts. Additional analyses examined content type (static posts vs reels), user status (followers vs nonfollowers), and audience activity patterns. These objective metrics complemented the self-reported survey data by providing an additional measure of user engagement.

### Qualitative Analysis

From a qualitative perspective, the study aimed to better understand students’ lived experiences and perceptions of learning with the Histo.Embryo Instagram account. Open-ended survey responses and informal exchanges during practical sessions were used to explore the perceived benefits and limitations of the tool, to identify barriers and facilitators to its use, and to capture students’ impressions of the learning climate it helped to foster. Particular attention was paid to how the Instagram account may have influenced student motivation, engagement, and the perceived proximity between teachers and learners. The survey instrument was developed based on the questions we raised as teachers in our disciplines. It was then refined through informal discussions with our peers. As the survey was exploratory, no formal validation was conducted.

Thus, qualitative data were obtained through 2 main channels. First, the survey included open-ended questions that invited participants to share their individual experiences, perceived benefits, limitations, or suggestions regarding the Instagram account. Free-text responses were analyzed using an inductive approach. Responses were grouped thematically to identify recurring patterns. Second, informal feedback was gathered directly during laboratory sessions, where instructors engaged in spontaneous, unstructured discussions with students. Informal feedback was used as contextual qualitative insights rather than systematically analyzed qualitative data.

These in-person interactions provided a more nuanced understanding of how the Instagram posts were integrated into students’ learning routines and how the platform influenced their motivation, attention, and perceived sense of connection to the course material. Qualitative data were analyzed using a simple thematic approach. Responses were reviewed and grouped into recurring themes by the authors to identify common patterns in students’ perceptions and experiences.

### Ethical Considerations

Participation in this study was voluntary and anonymous. Before completing the survey, participants were informed about the study objectives, the anonymous nature of data collection, and the intended use of the data for research purposes. Completion of the questionnaire was considered to imply informed consent. Participants could discontinue participation at any time without consequence.

All survey responses were anonymized and analyzed in aggregate form. Instagram analytics were extracted exclusively in aggregated form using Instagram Insights and did not involve access to personal accounts, private content, or identifiable personal information. All images used in the Instagram posts originated from educational resources or were produced by educators and contained no identifiable patient information.

This study evaluated a national educational initiative through an anonymous voluntary survey, informal educational feedback, and aggregated Instagram analytics. It did not involve any intervention involving human participants, collection of identifiable health data, or any procedure modifying participants’ care or behavior. In accordance with the French Public Health Code governing research involving human participants (Code de la santé publique, Articles L1121-1), this educational evaluation was conducted in compliance with the applicable French regulatory framework. Accordingly, prior review by a Comité de Protection des Personnes (Committee for the Protection of Persons) was not required.

No compensation was provided to participants, and no identifiable individual information is reported in this manuscript.

## Results

### Social Media Use and Engagement

Among surveyed participants, Instagram emerged as the dominant social media platform, with nearly 90% (112/125) of students reporting multiple daily uses. This far outpaced other platforms such as Facebook, TikTok, or Snapchat, which were used with notably less frequency (Table 1), confirming the platform’s strong embeddedness in students’ daily digital routines (Table 1). These usage patterns confirm that Instagram holds a central place in students’ online behavior, offering a potentially effective medium for delivering educational content. The habitual nature of its use among health sciences students suggests it may serve as a natural extension of their informal study practices, particularly when aligned with their academic needs.

**Table 1.** Frequency of social media platform usage among respondents of the online questionnaire (N=125).

Responses	Respondents, n (%)
Distribution of social media platforms use among student respondents	
Instagram	125 (100)
Facebook	75 (60)
TikTok	31 (25)
Snapchat	7 (6)
Other	7 (6)
Frequency of Instagram use among student respondents	
Several times per day	101 (81)
Once per day	11 (9)
Few times per week	8 (6)
Rarely	5 (4)

### Platform Engagement Metrics and Usage Patterns

Analysis of Instagram’s built-in analytics provided descriptive insights into the use of the Histo.Embryo Instagram account. Over the most recent 90-day period, the account recorded a total of 168,778 views, with 76.3% (128,778/168,778) of these views coming from followers and 23.7% (40,000/168,778) from nonfollowers, indicating a consistent and loyal student audience. During the same period, 2494 individual accounts were reached (Figure 2A). Analysis of content format showed a strong preference for static, image-based posts, which accounted for 98.1% (203/207) of all views. In contrast, Reels represented only 1.9% (4/207) of total content consumption (Figure 2B). However, these data should be interpreted with caution, as very few Reels were published during the study period, reflecting the predominantly image-based editorial strategy of the account. Active engagement metrics reflected similar trends: 979 interactions (likes, shares, saves, and comments) were recorded, of which 97.5% (954/979) came from followers. A total of 305 unique accounts interacted with content, suggesting that the account primarily served an engaged, follower-based educational community (Figure 2C). The most viewed posts were histology-based quizzes and image identification exercises, with the top-performing publications receiving between 6200 and 6500 views (Figure 2D). These data suggest that visually focused, question-driven formats generate the highest levels of attention and interaction.

**Figure 2.**
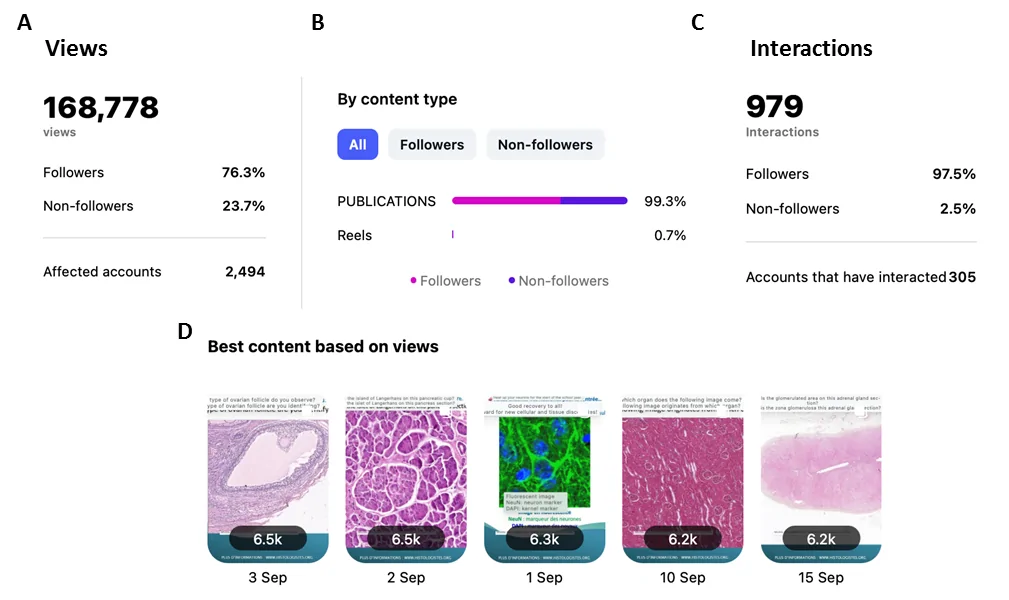
Instagram engagement metrics for the Histo.Embryo Instagram account over the last 90 days. (A) Total number of views were 168,778, with 76.3% (128,778/168,778) from followers and 23.7% (40,000/168,778) from nonfollowers, reaching 2494 unique accounts. (B) Distribution of views by content type: 98.1% (203/207) from standard image-based posts and only 1.9% (4/207) from Reels. (C) Engagement metrics showing 979 total interactions (likes, comments, shares, and saves) from 305 individual accounts, with 97.5% (954/979) of interactions from followers. (D) Top-performing posts based on views, all histology-based quizzes, each receiving between 6200 and 6500 views.

Survey data appeared to support this pattern of sustained engagement. Over one-third of respondents (48/125, 38%) had been following the account for more than a year, and an additional 29 % (36/125) for 6 to 12 months. Only 15% (19/125) had followed it for less than 3 months, indicating that the majority were not new users (Table 2). Patterns of engagement frequency revealed diverse engagement behaviors. Approximately one-third of respondents accessed the account multiple times per week, another third less than once a month, and the remainder consulted it either weekly (14/125, 11%) or a few times per month (29/125, 23%) (Table 2). These heterogeneous patterns indicate that the platform supports both regular and occasional use, depending on students’ study habits and needs.

These observations suggest that visually focused, question-driven formats may attract higher levels of attention and interaction.

**Table 2.** Patterns of student engagement with the histo.embryo Instagram account among respondents of the online questionnaire (N=125).

Responses	Respondents, n (%)
Duration of student engagement with the Histo.Embryo Instagram account among student respondents	
More than a year	48 (38)
6 to 12 months	36 (29)
3 to 6 months	22 (18)
Less than 3 months	19 (15)
Frequency of consultation of Histo.Embryo Instagram posts among student respondents	
Several times per week	41 (33)
Once a week	14 (11)
Few times per month	29 (23)
Less than once per month	41 (33)

### Perceived Usefulness of Content and Student Preferences

Survey responses indicated that MCQs were by far the most valued content type published on the Histo.Embryo Instagram account. A total of 88% (110/125) of students identified MCQs as particularly helpful for their learning (Table 3). Other highly appreciated formats included visual exploration tools, such as the “zoom in and zoom out” feature, and posts explaining methods for studying histological structures. Content related to embryology and cytogenetics, although generally well-received, was mentioned less frequently, suggesting a more limited perceived utility or possibly lower visibility within the account’s publication history.

**Table 3.** Perceived usefulness of published content and student preferences for future Instagram posts among respondents of the online questionnaire (N=125).

Responses		Respondents, n (%)
Most useful content types for learning among student respondents		
Multiple-choice quizzes		110 (88)
Zoom in and zoom out		76 (61)
Study methods for tissues		59 (47)
Embryology		21 (17)
Cytogenetics		9 (7)
None or N/A^a^		2 (2)
Most requested content types for future posts among student respondents		
Comparisons between normal and pathological histology		106 (85)
Annotated images		100 (80)
Multiple-choice quizzes		80 (64)
More detailed textual explanations		44 (35)
Video formats		32 (26)

a N/A: not applicable.

When asked about desired future content, students most commonly requested comparative posts between normal and pathological histology. Annotated images and additional MCQs were also frequently cited (Table 3). Beyond these, many respondents expressed interest in more detailed textual explanations and video-based formats, reflecting a desire for greater depth and multimodal engagement.

### Perceived Educational Impact of the Histo.Embryo Instagram Account

Survey results demonstrated a strong positive reception of the Histo.Embryo Instagram account among students. A total of 98% (122/125) of respondents reported that the account improved their understanding of histology, embryology, and cytogenetics to varying degrees. Among them, 38% (47/125) indicated a strong improvement, 33% (41/125) a moderate one, and only 2% (3/125) reported no perceived benefit (Table 4).

The account’s educational utility extended beyond passive consumption. When asked whether they had used information from the posts in academic settings, 50% (63/125) of students stated they had done so once or twice, and an additional 16% (20/125) had used it several times, such as during lectures, tutorials, or exams (Table 4). This indicates that the platform not only disseminates information but also supports practical knowledge retrieval and application.

**Table 4.** Perceived educational impact of the Histo.Embryo Instagram account among respondents of the online questionnaire (N=125).

Response	Respondents, n (%)
Perceived impact of the Histo.Embryo account on students’ understanding by student respondents	
A lot	47 (38)
Moderately	41 (33)
A little	34 (27)
Not at all	3 (2)
Reuse of Instagram content in academic settings	
Yes, several times	20 (16)
Yes, once or twice	63 (50)
No, never	42 (34)
Perception of improved ability to recognize histological structures	
A lot	36 (29)
Moderately	44 (35)
A little	41 (33)
Not at all	4 (3)
Perceived impact of Instagram content on student memorization	
A lot	25 (20)
Moderately	46 (37)
A little	43 (34)
Not at all	11 (9)

Regarding visual pattern recognition, a central skill in histology, 64% (80/125) of students reported a moderate to strong improvement after engaging with the content (Table 4). Additionally, 37% (46/125) reported that the posts moderately supported memorization of key concepts, while 34% (43/125) felt a slight improvement, and 20% (25/125) noted a strong effect (Table 4). These results highlight the role of the account in reinforcing cognitive skills essential for morphology-based learning.

### Qualitative Feedback and Suggestions

The responses to the open-ended questions of the survey are shown in Table 5, and informal feedback in Table 6. Free-text comments were analyzed using an inductive descriptive approach. Responses were grouped into themes by the authors to identify recurring patterns. Representative examples were selected to illustrate each theme; no formal qualitative coding or quantification was performed. Qualitative responses underscored the perceived relevance, usefulness, and pedagogical value of the Histo.Embryo Instagram account. Students widely appreciated the initiative, describing it as “in step with current trends” and “aligned with their digital habits,” and viewed the platform not as a superficial add-on but as a meaningful extension of their formal learning environment. Many respondents highlighted that the account helped them build visual recognition skills, strengthen conceptual understanding, and maintain a form of “general histology culture” between practical sessions. The clarity of explanations and high quality of histological images were frequently praised, with several students voluntarily expressing gratitude for the work behind the posts.

**Table 5.** Student responses regarding Instagram improvement and use of other online resources.

Question and theme	Description	Illustrative examples
Question 1: do you have any other suggestions to improve the Instagram account?		
Content format diversification	Requests to diversify formats to enhance comprehension and engagement	Add explanatory videos Include more posts with captions Incorporate animations with multiple-choice quizzes in stories
Interactivity enhancement	Desire for more active learner engagement	Increase interactivity Provide regular review quizzes
Pedagogical structuring of content	Need for better organization and sequencing of content	Create thematic series to structure the content Offer posts with case-based learning
Platform transparency	Interest in understanding how the account is managed	Clarify how many people manage the account
Positive feedback	Expressions of appreciation without specific suggestions	Thank you very much for creating this page
Question 2: do you use other online resources to supplement your learning?		
Video-based learning resources	Use of audiovisual materials for conceptual understanding	YouTube tutorials explaining microscopy slides
Structured educational platforms	Use of dedicated medical learning platforms	Osmosis University-provided e-learning resources
Visual reference resources	Use of image-based materials for recognition and memorization	Google Images Digital atlases of histology
Active learning tools	Use of tools supporting memorization and self-testing	Flashcard applications (eg, Anki)
Text-based academic resources	Use of written materials for formal learning	Lecture notes provided by professors Studies from scientific societies
Interactive and community-based resources	Use of platforms enabling discussion or applied learning	Discussion forums for medical students specialized mobile applications
Supplementary educational media	Use of alternative formats for reinforcement	Podcasts on medical education

**Table 6.** Inductive thematic grouping of free-text student responses with illustrative examples.

Theme	Description	Illustrative examples
Content frequency expectations	Requests for increased posting frequency, particularly during high-demand periods	A desire for more frequent posts, especially around exam periods
Visual enhancement of content	Demand for more visually engaging learning materials	Requests for more visually engaging content such as infographics
Clinical integration	Interest in strengthening links between basic sciences and clinical application	Suggestions to strengthen links between histology and clinical practice
Active learning strategies	Requests for tools supporting self-assessment and active recall	A call for more quizzes to support active recall and self-assessment
Perceived usefulness and satisfaction	Positive feedback regarding the initiative’s value	Overall satisfaction with the initiative, highlighting its usefulness as a complementary tool
Content frequency expectations	Requests for increased posting frequency, particularly during high-demand periods	A desire for more frequent posts, especially around exam periods

A large majority reported combining Instagram content with traditional course materials rather than relying exclusively on either format. Instagram was seen as a complementary and accessible tool for spaced repetition and microlearning, particularly effective during revision phases. The idea of a more interactive version of the account, featuring pedagogical stories, live sessions with instructors, or real-time quizzes, was met with high enthusiasm. Students often recommended the account to their peers, reinforcing its status as a peer-validated resource.

Suggestions for improvement coalesced around two primary themes: interactivity and content organization. Many students expressed a desire for increased interactivity through features such as quizzes in Instagram Stories, animations, or live questions and answers (Q&A) sessions with instructors. Others proposed structuring content by organ system or academic year, using color-coded formats to facilitate retrieval and navigation. There were also requests for more frequent posting, especially around exam periods, to support spaced learning and help reinforce core concepts through repetition in diverse contexts.

In open-ended survey questions, students also mentioned using a wide range of other digital resources for studying histology, including online atlases, YouTube videos (Google LLC), Osmosis (Elsevier), ChatGPT (OpenAI), Google Images (Google LLC), and institutional platforms. These responses highlight the multiplicity of learning tools students combine and suggest that Instagram fits into a broader, student-driven ecosystem of multimodal and asynchronous study aids.

Finally, informal feedback gathered during in-person histology laboratory sessions echoed these findings: students responded positively and enthusiastically when the Instagram account was referenced in class. They perceived it as a genuine and innovative effort to bridge the gap between traditional teaching and contemporary learning habits.

## Discussion

### Principal Findings

This study highlights the pedagogical potential of Instagram as a complementary teaching tool in morphology-based disciplines such as histology, embryology, and cytogenetics. Through a mixed methods design integrating analytics, survey responses, and qualitative feedback, our findings provide an integrated perspective on how social media can support student learning when aligned with learner habits and cognitive strategies. This is particularly relevant in histology, a discipline that relies heavily on visual pattern recognition and image-based learning, making it especially well-suited to platforms such as Instagram [2]. Importantly, engagement metrics such as views, reach, or interactions should not be interpreted as direct indicators of learning outcomes. While they reflect content exposure and user activity, they do not capture the depth of cognitive processing or actual knowledge acquisition, as highlighted in prior research on digital learning environments [20].

Our results reinforce previous findings that Generation Z medical students are drawn to mobile-native, visually rich platforms that support asynchronous, self-directed learning [16,19,21]. Instagram’s intuitive design and ubiquity in students’ daily routines provide low-barrier access to educational content. Recent studies have further emphasized the growing role of Instagram as a structured educational tool within medical curricula, supporting learner engagement and fostering online learning communities [22]. By embedding concise, image-based posts into platforms students already use regularly, the Histo.Embryo Instagram account facilitated voluntary engagement in informal settings, reflecting digital pedagogy trends that emphasize learner autonomy, personalization, and cognitive alignment [1,4,23]. These learning behaviors align with principles of “microlearning” and “just-in-time” learning, which deliver targeted content in short intervals (seconds to minutes) and have been shown to enhance retention and knowledge transfer [11,19]. The account’s asynchronous structure allowed students to revisit content on their own terms, promoting autonomy and repeated exposure, particularly useful in image-based learning domains.

Student feedback suggested that the account was not perceived as a superficial add-on, but rather as a meaningful extension of formal teaching. Many participants reported using it to reinforce concepts, enhance pattern recognition, and retrieve visual knowledge during practicals or assessments. The Histo.Embryo Instagram account thus functioned as a reusable visual study library, consulted both routinely and strategically. Indeed, while Instagram offers valuable opportunities for engaging students through concise, visually oriented content aligned with their digital habits, its structural limitations constrain its use as a fully systematic learning resource. In particular, challenges related to content organization, navigation, and retrieval may hinder its effectiveness for comprehensive or sequential learning. These limitations are further reflected in students’ requests for improved content structuring. Although certain platform features, such as highlights or thematic grouping, can partially enhance accessibility, they remain insufficient to replicate the coherence and progression of formal curricula or dedicated educational platforms. Therefore, this initiative is better understood as a complementary tool, supporting revision and reinforcement of key concepts, rather than as a standalone learning environment. This sustained and strategic use aligns with findings from recent studies in histology education, which demonstrate that Instagram-based learning activities can enhance engagement, motivation, and even academic performance, particularly when built around image-based questions and interactive formats [2]. These findings are further supported by interventional studies demonstrating that social media–based educational approaches can lead to measurable improvements in knowledge acquisition, with postintervention scores increasing substantially following structured video-based learning initiatives [24]. In this context, the account can be understood as a reusable visual study library, consulted both routinely and strategically. Such longitudinal integration into students’ study practices suggests that educational Instagram accounts, when intentionally designed, can become a meaningful component of the informal learning ecosystem in medical education [6,16]. Preferences for static image-based posts, particularly MCQs and “zoom in and zoom out” formats, support previous evidence that stepwise visual scaffolding and active recall are effective strategies for durable learning [19]. The swipe-based carousel design mimics flashcard dynamics, prompting reflection before answer disclosure and promoting cognitive engagement [1]. These findings are consistent with previous work showing that students preferentially engage with practical, memory-oriented, and clinically relevant content on Instagram, highlighting the importance of aligning digital resources with learner expectations [9]. Together, these results underscore the value of combining visual scaffolding with active learning principles in digital environments.

Suggestions for improvement focused on increased interactivity and improved content organization. Students requested features like Story-based quizzes, animations, live Q&A sessions, and clearer categorization by organ system or academic level. These align with established best practices in digital pedagogy that recommend multimodal delivery and cognitive scaffolding [4,6]. Instagram Stories, in particular, offer opportunities for real-time, anonymous feedback and may further support low-stakes learning engagement [1,7]. The demand for comparative histology posts (normal vs pathological) also suggests a desire to integrate foundational science with clinical reasoning early in medical education. Visual formats that facilitate contrast and comparison can support diagnostic thinking and are well-suited to Instagram’s format [9]. While memorization benefits were rated more modestly, students noted that mechanisms like visual repetition and dual coding supported their learning [4]. These approaches are known to support understanding and retention, especially when spaced repetition is embedded within digital tools. Even passive exposure may reinforce core concepts over time [1].

Beyond cognitive benefits, qualitative feedback underscored the emotional and relational value of the initiative. Students emphasized the clarity of explanations, the quality of images, and the perceived pedagogical investment of educators, describing the account as aligned with their learning habits and expressing a sense of pedagogical trust and gratitude. These socioaffective dynamics, rarely captured by analytics alone, further validate the educational value of well-designed social media platforms. This community dimension has been described in broader contexts, where Instagram facilitates the dissemination of scientific knowledge and the development of knowledge-sharing networks among users [25].

At the same time, students also reported using other digital resources in parallel, including histology atlases, YouTube, Osmosis, ChatGPT, and institutional platforms, reflecting the multiplicity of tools in contemporary student learning ecosystems. Previous studies have reported that, despite recognizing the benefits of social media for learning and communication, learners may feel overwhelmed by the need to manage several platforms simultaneously, highlighting the importance of coherent and structured pedagogical integration [26]. Within this landscape, Instagram occupies a distinct niche: a visually anchored, pedagogically consistent, and socially embedded learning tool that bridges formal instruction and informal exploration.

Taken together, these findings support the potential value of educational Instagram accounts like Histo.Embryo Instagram account as complementary tools in medical education. When designed intentionally and supported institutionally, such platforms can strengthen visual literacy, promote learner autonomy, and extend the reach of formal instruction into digital environments where students are already actively engaged.

### Reflections on the Impact of the Instagram Account on Teachers

This section reflects the authors’ perspectives and experiences as educators involved in the project, rather than data derived from a formal evaluation. The perceived benefits for teachers described in this section are based on reflective insights from the authors and participating educators, rather than on systematically collected empirical data. It is important to emphasize the beneficial impact of the Instagram account from the perspective of the educators involved in the project. The process of content creation represented a demanding yet highly rewarding endeavor for us as teachers, with significant benefits on both pedagogical and professional levels.

From a pedagogical standpoint, this initiative allowed for the regular updating of course material, integrating the most recent knowledge, official recommendations, and new digital resources. It also provided an opportunity to adapt teaching delivery to the needs of students, taking into account their learning habits and their relationship with digital tools. In this sense, the design of Instagram posts fostered pedagogical innovation, offering a dynamic complement to traditional methods. The act of designing content further stimulated critical reflection on teaching practices and served as a lever for the development of teaching skills. The preparation of Instagram posts also contributed to the enhancement of academic and disciplinary expertise, reinforcing recognition from students, peers, and the institution. Moreover, this process renewed the teacher’s motivation, preventing routine and sustaining professional enthusiasm over time.

Beyond the individual scale, the benefits also extended to the institutional and societal levels. The development of new pedagogical content contributed to the harmonization of teaching practices in line with national and international standards, while simultaneously strengthening the attractiveness of our disciplines through innovative, interactive, and engaging methods. By preparing students with updated and adapted content, educators also addressed the needs of the health care system by training competent professionals capable of integrating scientific and pedagogical advancements into their future practice.

Thus, the creation of new content emerged as both a demanding and enriching process, associated with high levels of student engagement and perceived usefulness. While these findings suggest that such initiatives may support learning and align with students’ digital practices, they primarily reflect patterns of content consumption and perceived value rather than direct evidence of educational effectiveness. As such, this approach should be considered a complementary educational tool, whose impact on learning outcomes warrants further investigation.

### Limitations and Future Research Directions

While this study explores the use of Instagram as a complementary educational tool in morphology-based learning, several limitations warrant consideration. First, our evaluation primarily relied on self-reported perceptions of self-reported perceived usefulness for learning, rather than objective academic performance metrics such as exam scores. Although a substantial proportion of students reported using the Instagram content during tutorials or assessments, this does not allow us to establish a causal relationship between Instagram use and academic success, a limitation echoed in prior studies [1,7]. The study design may also be subject to self-selection bias, as participation in both the Instagram activity and the survey was voluntary, potentially attracting more motivated or engaged students. In addition, responses may have been influenced by social desirability bias, as the initiative was actively promoted by instructors involved in teaching. Furthermore, respondents were not formally verified as enrolled students, which may limit the specificity of the sample. Finally, the survey instrument was developed for this study and was not formally validated, and the findings therefore reflect exploratory insights into students’ perceptions rather than standardized measures of learning. Future research using controlled study designs comparing students who use educational Instagram accounts with matched non-user cohorts would be necessary to assess the platform’s true student engagement with educational content.

Second, although the account was public and regularly promoted in class, visible interaction rates (likes, comments, and shares) remained modest relative to total reach. This suggests a primarily passive engagement model, where students consume content without interacting publicly, a behavior consistent with previous findings on unilateral social media use in education [5,9]. This interpretation is supported by the Platform Engagement Metrics, which show a marked discrepancy between high reach and impressions and relatively low levels of visible interactions (likes, comments, and shares), suggesting that most users engaged with the content through viewing rather than active participation. We did not investigate whether this limited interaction stemmed from platform discomfort, uncertainty about how to engage, concerns about public visibility, or just a lack of interest. Familiarity with Instagram for personal use does not necessarily translate into pedagogical engagement [9].

Third, the rapidly evolving nature of social media platforms raises concerns about sustainability. While Instagram currently enjoys widespread student adoption, user preferences can shift quickly, as shown in previous studies [5,27]. What is pedagogically effective today may lose relevance in the near future, challenging long-term integration into curricula. Additionally, engagement metrics drawn from Instagram analytics may include interactions from nonstudent users, complicating efforts to isolate educational behaviors within the target population. Furthermore, interaction metrics were analyzed in aggregate form, as detailed historical data (eg, likes, shares, comments, and saves by content type) were not fully accessible retrospectively beyond the platform’s standard analytics window. This limitation restricts a more granular interpretation of engagement patterns across post formats. In addition, content performance could not be normalized on a per-post basis, as post-level metrics and the exact distribution of posts across formats were not systematically recorded.

Fourth, no formal onboarding or training was provided to guide students in using the platform for academic purposes. According to the Salmon model of online learning, structured induction is essential to transition learners from passive content consumption to deeper engagement and knowledge construction. In the absence of such scaffolding, students may have remained at the lower levels of digital engagement, using Instagram primarily as a content consumption tool rather than an interactive learning space [28]. More broadly, the educational use of social media raises important pedagogical considerations regarding the need for critical engagement and structured guidance. Without appropriate framing, these platforms may remain tools for passive consumption rather than deeper learning, highlighting the importance of their integration within a coherent pedagogical approach [29].

In addition to these pedagogical constraints, broader behavioral and psychosocial risks associated with social media use must be acknowledged. Prior studies have reported negative effects such as digital distraction, blurred boundaries between academic and personal life, and risks of cyberbullying, privacy concerns, or social comparison. Furthermore, social media interventions may not reach students who are less digitally active and can contribute to digital fatigue in some cases.

These limitations point to important directions for future research. Studies that combine subjective perceptions with academic objective learning outcomes, explore engagement across different platforms and formats, and assess the impact of structured onboarding could help refine the design of social media–based pedagogical interventions. Investigating long-term retention, cross-platform engagement patterns, and student motivation in digital learning environments will be particularly valuable in optimizing these tools for health education.

### Conclusions

This study demonstrates that Instagram, when used intentionally and aligned with pedagogical principles, can serve as a valuable adjunct to traditional teaching in morphology-based medical education. The Histo.Embryo Instagram account initiative successfully engaged students through visually rich, accessible, and interactive content, supporting pattern recognition, concept reinforcement, and just-in-time learning. However, the limitations related to self-reported outcomes, passive engagement, and platform sustainability highlight the need for cautious interpretation. Future research should seek to correlate social media use with academic performance and explore how structured onboarding and evolving digital tools can further enhance student engagement and learning outcomes.

## Abbreviations

CHEC: College of Histologists, Embryologists, and Cytogeneticists
MCQ: multiple-choice quiz
Q&A: question and answer
